# The Diamesinae (Diptera: Chironomidae) in High‐Altitude Andean Streams Using Morphological and Molecular Approaches

**DOI:** 10.1002/ece3.72333

**Published:** 2025-11-27

**Authors:** Diana C. Hoyos Jaramillo, Raúl Acosta, Carles Ribera, Núria Bonada, Narcís Prat

**Affiliations:** ^1^ FEHM‐Lab (Freshwater Ecology, Hydrology and Management), Departament de Biologia Evolutiva, Ecologia i Ciències Ambientals, Facultat de Biologia Universitat de Barcelona (UB) Barcelona Spain; ^2^ Fundación FUNMAJO, EBA, RAIEC, Biodiversity Branch Boyacá Colombia; ^3^ Institute of Environmental Assessment and Water Research (IDAEA), CSIC, SHE2 (Surface Hydrology, Ecology and Erosion), FEHM‐Lab (Freshwater Ecology, Hydrology and Management) Barcelona Spain; ^4^ Departament de Biologia Evolutiva, Facultat de Biologia, Ecologia i Ciències Ambientals Universitat de Barcelona Barcelona Spain; ^5^ Institut de Recerca de la Biodiversitat (IRBio) Universitat de Barcelona (UB) Barcelona Spain

## Abstract

Diamesinae is one of the 11 subfamilies of Chironomidae, characterized by its preference for cold waters, typically found in mountainous regions. We aimed to study the biodiversity and distribution of two genera of this subfamily found in high‐altitude streams in the Andes (*Paraheptagyia* Brundin, 1966 and *Limaya* Brundin, 1966), by combining morphological and molecular analyses. A database with 190 larval specimens of Diamesinae (160 of *Paraheptagyia* and 22 for *Limaya*), collected between July and October of 2011 from 20 streams in Colombia, Ecuador, and Peru, at altitudes above 2000 m a.s.l. was used. *Paraheptagyia* was found in the three countries, whereas *Limaya* was only found in Ecuador and Peru. Morphological analyses were based on 105 larvae mountings, using several measurements (26 measurements for *Limaya* and 25 for *Paraheptagyia*) and specimens for each morphotaxon. As a result, the head capsule width and the antennal ratio (AR) were the most valuable differentiators to distinguish between two morphotypes of *Limaya*, whereas *Paraheptagyia* could not be distinguished by using morphological characters. We employed the ABGD, ASAP, and bPTP methods, and supported them with distance matrices and haplotype networks, to delineate operational taxonomic units (OTU) based on 130 molecular sequences (113 for *Paraheptagyia* and 17 for *Limaya*). For *Limaya*, we identified two OTUs with all methods, one from Ecuador and one from Peru. As for *Paraheptagyia*, we identified between seven and nine OTUs, of which we found the majority in Peru, and two occurred in both Ecuador and Colombia. We hypothesize that the presence of the Huancabamba depression on the border between Ecuador and Peru likely acts as a genetic barrier, limiting gene flow. The difference in branch length between Peruvian and Ecuadorian/Colombian species supports Brundin's (1966) hypothesis of a south‐to‐north colonization pattern and the Gondwanan origin of both genera.

## Graphical abstract

Diamesinae is one of the 11 subfamilies of Chironomidae, characterized by its preference for cold waters, typically found in mountainous regions. For the genus *Limaya*, two OTUs were identified using all methods, one for Ecuador and one for Peru. Seven OTUs were identified for the genus *Paraheptagyia*. Five of the seven OTUs were found in Peru, and the other two were shared between Ecuador and Colombia.

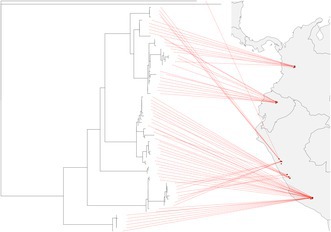

## 1 Introduction

Chironomidae is a poorly studied family of Diptera in South America. In recent years, taxonomic studies in Brazil and Argentina have made some substantial contributions to the understanding of this group, followed by research in Colombia, Ecuador, and Peru (Acosta and Prat 2018; Donato et al. 2023; Silva et al. 2023; Hoyos‐Jaramillo and Gomes‐Dias 2020; Trivinho‐Strixino 2011). Beyond their ecological significance, Chironomidae of South America have played a key role in understanding the effects of plate tectonics on speciation. Their antiquity, evolutionary plasticity, high diversity, and relatively low vagility make them valuable models for biogeographic studies (Brundin 1966; Ferrington 2008; Silva and Ekrem 2016; Silva et al. 2023). For example, based on Chironomidae and subfamilies such as Diamesinae, Brundin (1966) proposed a Gondwanan dispersal pathway to explain the phylogenetic similarities among species from Australia, New Zealand, South America, and South Africa. This hypothesis has been further supported by molecular phylogenetic studies at the family, subfamily, and genus levels (Cranston et al. 2010, 2012; Lin et al. 2018; Krosch et al. 2011, 2020; Krosch and Cranston 2013).

One of the 11 recognized subfamilies of Chironomidae, the Diamesinae, was long considered a group within the subfamily Orthocladiinae, even after Edwards (1929) separated it as a subfamily and gave it that status. Brundin (1966) retained it in his original work as a group of Orthocladiinae. Saether (1977) restored the subfamily status in his work on phylogenetic systematics. Currently, the subfamily comprises six tribes, 22 genera, and about 100 species. They are mostly restricted to freshwater ecosystems with low temperatures (Brundin 1966), usually in rivers with high flows and in the upper parts of mountains, where temperatures are known to be lower.

**FIGURE 1:**
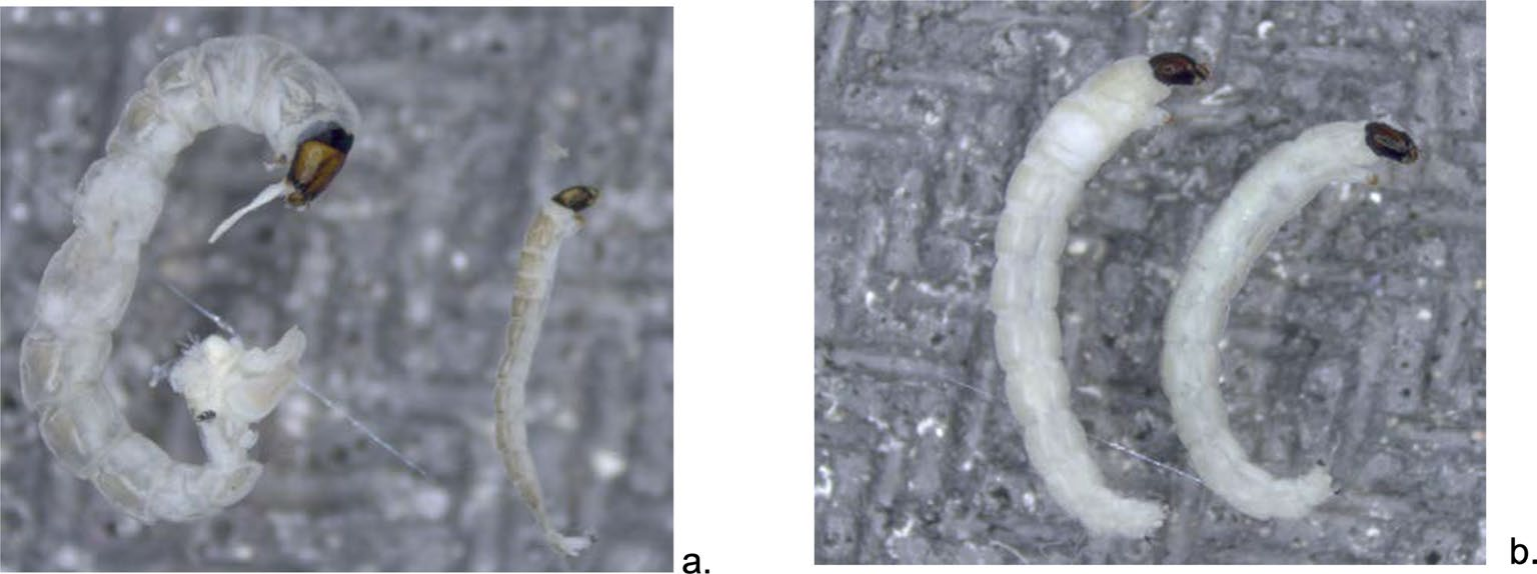
Representative Diamesinae specimens. (a) Two larvae of *Paraheptagyia*, showing a mature stage and an early stage. (b) Two larvae of *Limaya*. Images adapted from the morphological key by Prat et al. (2018).

The lack of available information on the Diamesinae and their genera in South America is reflected in the limited number of published works on the subject (Brundin 1966; Roback and Coffman 1983; Prat et al. 2014, 2018). So far, there are five known genera of Diamesinae in South America: *Limaya* Brundin 1966 and *Paraheptagyia* Brundin 1966, distributed from Chile to northern South America, and *Heptagyia* Philippi 1865, *Mapucheptagyia* Willassen 1995 and *Reissmesa* Ashe 2000 that are restricted to the southern region of South America. Brundin's work in 1966 initially reported the distribution of genera *Limaya* and *Paraheptagyia* from Patagonia to Ecuador. Later, Roback and Coffman (1983) recorded both genera from the Bolivian‐Peruvian Altiplano and the Venezuelan Páramo, thus extending the known distribution of *Paraheptagyia* to northern South America. Finally, Ruiz‐Moreno et al. (2000) reported the genus *Paraheptagyia* for Colombia, confirming its presence in the northern Andes. Representative images of larvae from these genera are shown in Figure 1. Although subsequent studies have successfully identified both genera of Diamesinae and expanded their known distribution (Paggi 2009; Acosta and Prat 2010; Prat et al. 2014, 2018), a comprehensive understanding of their full diversity, ecology, and biogeography in the region remains incomplete, underscoring the need for continued research to deepen our knowledge of these genera.

In 1966, Brundin established *Paraheptagyia* as a genus based on South American specimens, designating *Heptagyia cinerascens* Edwards 1931, as the type species of the genus. *H. cinerascens* was first described by Edwards (1931) from a few specimens found in Patagonia and is now documented in Ecuador, Peru, Argentina, and Chile. Brundin also included two other species described by Edwards (1931) in the genus: *Paraheptagyia nitescens* and *P. semiplumata*, both with holotypes from Chile and also recorded in Argentina. Additionally, he incorporated *Paraheptagyia tasmaniae* (holotype from Tasmania, with current distribution restricted to the same region) and *Paraheptagyia tonnoiri* (holotype from Australia, also found in New South Wales), both of which were described by Freeman 1961. Later, Brundin (1966) formally added two more species to the genus: *Paraheptagyia umbraculata*, from Chile, and *Paraheptagyia andina*, described from Bolivia. Currently, the genus *Paraheptagyia* includes seven recognized species with distributions spanning South America, Australia, and Tasmania. With an emphasis on adult male taxonomy for the description of species, for many described *Paraheptagyia* species, adult females and immatures are unknown. Of the seven species of *Paraheptagyia*, four have complete descriptions (larvae, pupae, males, and females), two ( *P. nitescens* and *P. andina* ) have descriptions of males and pupae, and *P. umbraculata* was described from a single male (Brundin 1966). Available information from molecular studies is scarce, only the partial COI‐3PmtDNA sequence of *Paraheptagyia tonnoiri*, found in Australia (Cranston et al. 2010), in GenBank and the BOLD system, limiting its use in molecular investigations of the genus.

Brundin (1966) described *Limaya longitarsis*, the genus's only named species and holotype, using specimens from all life stages from Argentina. The species also has a known distribution in Chile. A second species (*Limaya* cf. *junin*, from Peru) of this genus, based on pupal description with notable differences from those of *L. longitarsis* , awaits its status when adult males are known (Brundin 1966).

High‐altitude Andean rivers, located within diverse ecosystems such as páramo and Andean forests, are crucial biodiversity hotspots and essential water sources for both surrounding communities and nearby populations (Elias et al. 2009; Villamarín et al. 2020). It has been hypothesized that, due to the geographical characteristics and the extension of the Andean Mountain range, ecological diversification and speciation were favored (Elias et al. 2009; Merckx et al. 2015). This scenario suggests a potential process of speciation undergone by Diamesinae, particularly within the genera *Limaya* and *Paraheptagyia*, along the studied altitudinal and latitudinal gradient. Furthermore, it is expected that species richness may be higher south of the Huancabamba Depression (located between northern Peru and southern Ecuador) than in the northern part of the continent (comprising parts of Ecuador and Colombia), potentially supporting Brundin's hypotheses regarding the northward dispersion of Gondwanan species from Chile.

The aim of this work was to assess the biodiversity and distributional patterns of *Limaya* and *Paraheptagyia* (Diamesinae: Chironomidae) in high‐altitude streams in the Andes, from southern Peru to northern Colombia. To achieve this, we employed a combination of morphological and molecular (DNA barcoding) methods for species delimitation mostly on larvae. Specifically, this research aimed to: delimit distinct morphospecies based on morphological characters and identify molecular operational taxonomic units (OTUs) using mitochondrial DNA sequences, to evaluate their congruence and identify instances of cryptic diversity or taxonomic inconsistencies.

## 2 Materials and Methods

We used a database (Table 1) created by the FEHM‐Lab group during the development of the Biqura project in three South American countries, Colombia (reaches of five streams), Ecuador (reaches of six streams), and Peru (reaches of nine streams). It included the collected samples between July and October of 2011, the *COI‐5P*mtDna sequences, microscopic slide mountings of immature chironomids, physicochemical variables of water, and habitat indices such as the river habitat index (IHF) and the riparian vegetation index for tropical regions (QBR‐And) done by Acosta et al. (2009) (Appendix S1).

**TABLE 1:** The geographical characteristics of the sampling sites from Biqura (Colombia, Ecuador, and Peru) and Globios (Peru) projects.

Code	Country	Basin	Slope	River	*Paraheptagyia*	*Limaya*	Coordinates		Altitude	Ecosystem type
					*n*	*n*	Latitude	Longitude	(m a.s.l)	
CH02	Colombia Biqura	Cauca	Atlantic	Romerales	1	0	4.989444	−75.432278	2485	Andean Forest
CH03		Cauca	Atlantic	Bergel	6	0	5.104314	−75.373258	3408	Paramo
CA1		Cauca	Atlantic	Campolegrito	1	0	4.85542	−75.51049	2883	Andean Forest
GL01		Magdalena	Atlantic	Nevado 1	13	0	4.939606	−75.33879	4032	Paramo
GL02		Magdalena	Atlantic	Nevado 2	10	0	4.940979	−75.342282	4022	Paramo
GY01	Ecuador Biqura	Guayllabamba	Pacific	Antisana 1	14	13	−0.530524	−78.228146	3987	Paramo
GY04		Guayllabamba	Pacific	Saltana	1	0	−0.31644	−78.22025	3869	Paramo
GY05		Guayllabamba	Pacific	Warmihuayco	10	0	−0.28712	−78.24562	3652	Andean Forest
NA02		Napo	Atlantic	Papa03	8	0	−0.41204	−78.02792	2457	Andean Forest
NA03		Napo	Atlantic	Negra	10	0	−0.38303	−78.06645	2672	Andean Forest
NA04		Napo	Atlantic	Cachiyako	6	0	−0.38886	−78.20333	3712	Andean Forest
NA05		Napo	Atlantic	Atujlarca	7	0	−0.38781	−78.20329	3716	Paramo
NA06		Napo	Atlantic	Chalpi	1	0	−0.30131	−78.12900	3926	Paramo
CO01	Peru Biqura	Ocoña	Pacific	Huancarama	1	2	−15.129233	−72.914236	3278	Andean Forest
CO02		Ocoña	Pacific	Sumana	17	1	−15.061697	−72.680521	3470	Andean Forest
CO03		Ocoña	Pacific	Manantial S/N	11	7	−15.020492	−72.70125	3854	Paramo
CO04		Ocoña	Pacific	S/N	1	5	−14.98615	−72.712037	3640	Andean Forest
CO05		Ocoña	Pacific	Chinche	21	0	−14.904091	−72.642193	4486	Paramo
CO06		Ocoña	Pacific	Sayrusa	9	0	−14.908976	−72.638414	4470	Paramo
MO02^a^		Mosna	Atlantic	Pucavado	3	0	−9.681147	−77.226747	4173	Paramo
SA12		Santa	Pacific	Llullan	6	0	−9.032297	−77.768489	2746	Andean Forest
SA05		Santa	Pacific	Ishinca sobre Collon	3	1	−9.382365	−77.521871	3364	Andean Forest
GLCHI	Peru Globios	Chillón	Pacific	Tributary	3	0	−11.393151	−76.502136	3840	Paramo
GLCHI		Chillón	Pacific	Tributary	4	0	−11.409612	−76.552498	3449	Andean Forest
GLRI01		Rimac	Pacific	Tributary	3	0	−11.733299	−76.270157	3432	Andean Forest
GLPA01		Cañete	Pacific	Paccha	9	0	−11.936590	−76.102313	4541	Paramo
GLPA02		Cañete	Pacific	Paccha	1	0	−11.960663	−76.114890	4362	Paramo
GLPA02		Cañete	Pacific	Paccha	2	0	−11.994039	−76.116305	4173	Paramo
GLSA02		Santa	Pacific	Cojup	3	0	−9.402387	−77.384373	4538	Paramo
GLCHU		Churup/Santa	Pacific	Quillcayhuanca	1	0	−9.442284	−77.355163	4183	Paramo

^a^ Means some specimens of *Paraheptagyia* were found. However, these specimens did not amplify and were of the third larval stage, rendering them unsuitable for inclusion in the analysis.

### 2.1 Study Area and Sampling

**FIGURE 2:**
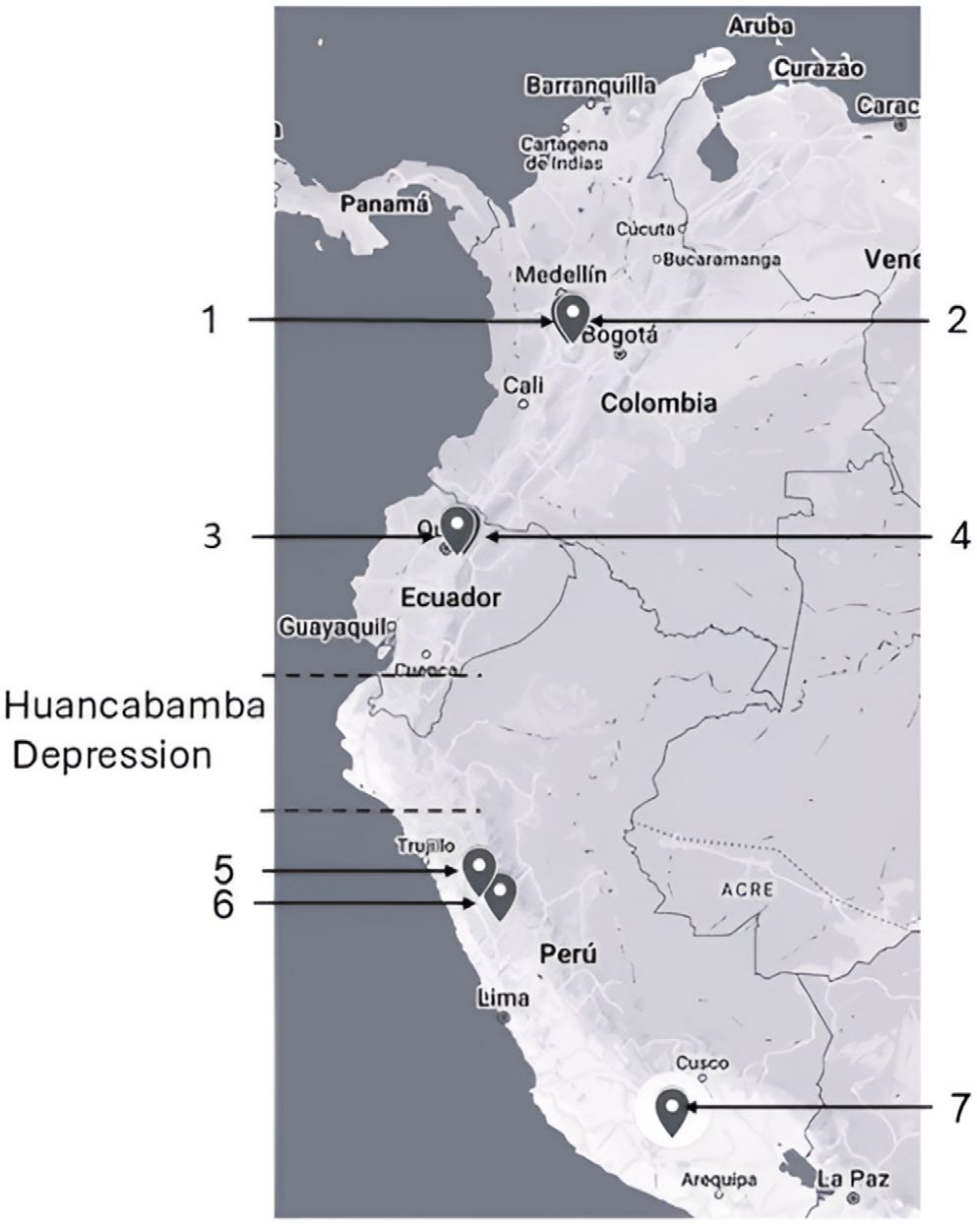
Map showing the location of the basins where Diamesinae specimens were collected in South America. Sampling locations are indicated by numbers: COLOMBIA (approx. 5° N) includes 1 Cauca and 2 Magdalena basins; ECUADOR (approx. 0°) includes 3 Guayllabamba and 4 Napo basins; PERU (approx. 10°–15° S) includes 5 Santa, 6 Mosna, and 7 Ocoña basins. Exact sampling point coordinates, geographical, and physicochemical characteristics are provided in Table 1 and Appendix S1.

The Biqura (https://www.fehm.cat/rios‐altoandinos/) sampling sites were located within high‐altitude mountain streams, spanning two distinct ecosystems: the Andean forest and the páramo, at elevations ranging from 2010 to 4670 m a.s.l. (Figure 2). In Colombia, the five streams were situated approximately at 5.0° N latitude; three flowed into the Cauca River Basin, and two entered the Magdalena River Basin. Both basins drained into the Atlantic Ocean. In Ecuador, six streams were located near the Equator (0.0° latitude), with four in the Napo River Basin (Atlantic drainage) and two in the Guayllabamba River Basin (Pacific drainage). In Peru, the nine streams occurred between 9.0° S and 15.0° S latitude. One stream, in the Mosna Basin, drained into the Atlantic, while the rest (two streams in the Santa Basin and six in the Ocaña Basin) drained into the Pacific.

The Globios project (Global observatory network for freshwater biodiversity in high mountain streams https://www.eucelac‐platform.eu/project/global‐observatory‐network‐freshwater‐biodiversity‐high‐mountain‐streams) provided an additional 26 sequences from Peru. These samples were collected between July and December of 2019 and May and July of 2021, from eight high‐altitude mountain streams, which span a range of altitudes from 3432 to 4541 m a.s.l. (Table 1) (Karen Velazquez, leg.).

The methodology for Biqura sampling, specimen preparation, and molecular analyses followed established protocols detailed in Epler (2001), Nuñez and Prat (2010), Prat et al. (2013, 2018), and Villamarín et al. (2013). The specimens analyzed in this study, along with their localities, mounting procedures, and molecular protocols, are summarized in Appendix S2. These resources provide a consistent framework for integrating morphological and molecular data in our analyses.

### 2.2 Morphometric Analysis

**FIGURE 3:**
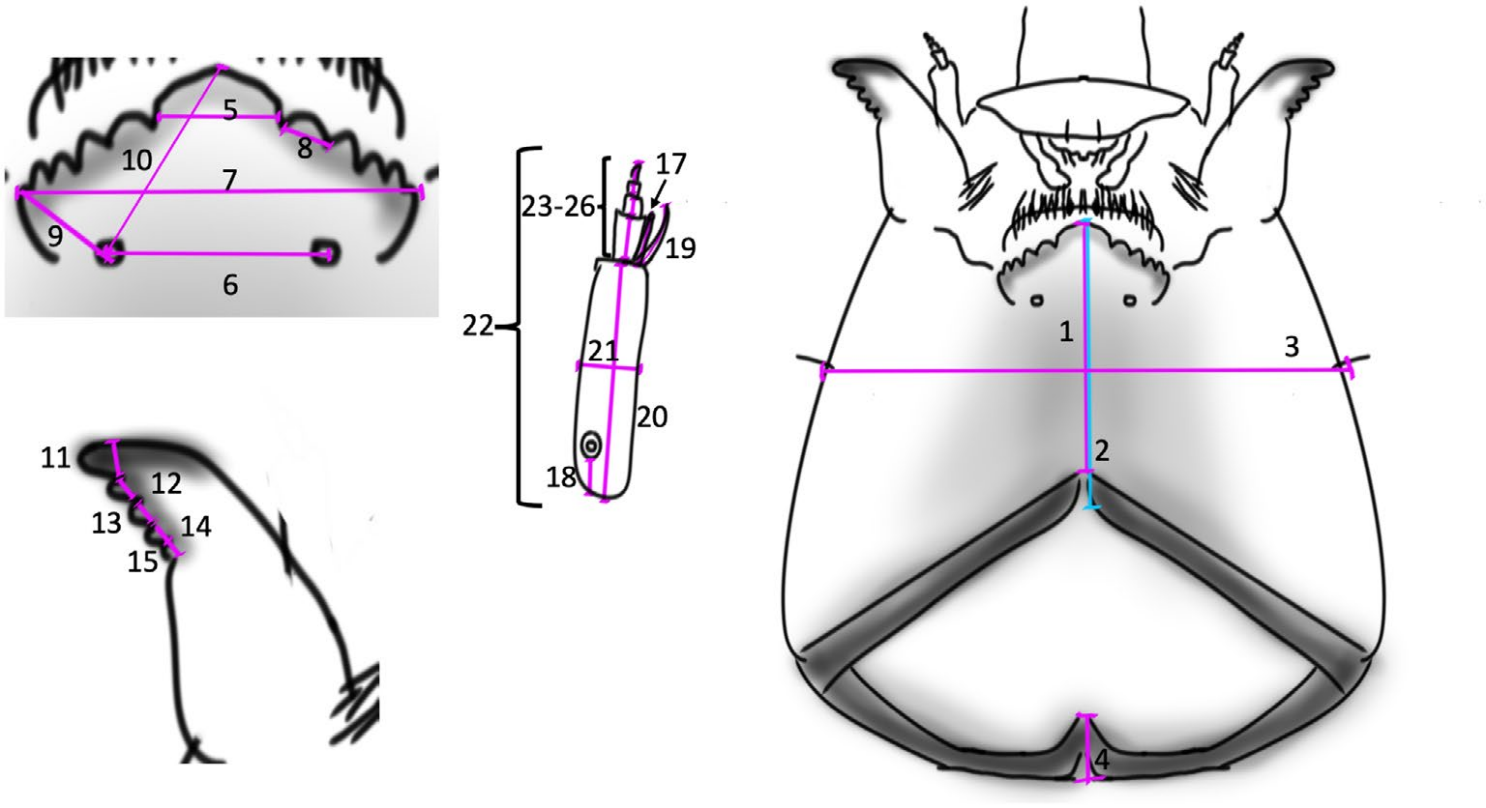
Morphological measurements evaluated for the *Limaya* larvae. Head Capsule:1 Length1: Measure from mentum to anterior post‐occipital margin. 2 Length 2: Measure from the mentum to the posterior occipital margin. 3 Width: Measure below the seta. Post‐occipital margin 4 Measure at the back of the post‐occipital margin. Mentum 5 Width of central tooth. 6 Length between seta submenta. 7 Length between tips of last lateral tooth. 8 Width of first lateral tooth. 9 Distance between last lateral tooth and seta submentalis. 10 Distance between central tooth and seta submentalis. 11 Width of the apical tooth. 12 Width of first internal tooth. 13 Width of second internal tooth. 14 Width of the third internal tooth. 15 Width of fourth internal tooth. Antenna 16 (antennal ratio): Length of the first segment divided by the sum of the lengths of the second to fifth segments. 17 Length of blade. 18 Distance between the base of the antenna to the OR. 19 Length of seta. 20 First segment length. 21 First segment width. 22 Total length. 23 Second segment length. 24 Third segment length. 25 Fourth segment length. 26 Fifth Segment Length.

**FIGURE 4:**
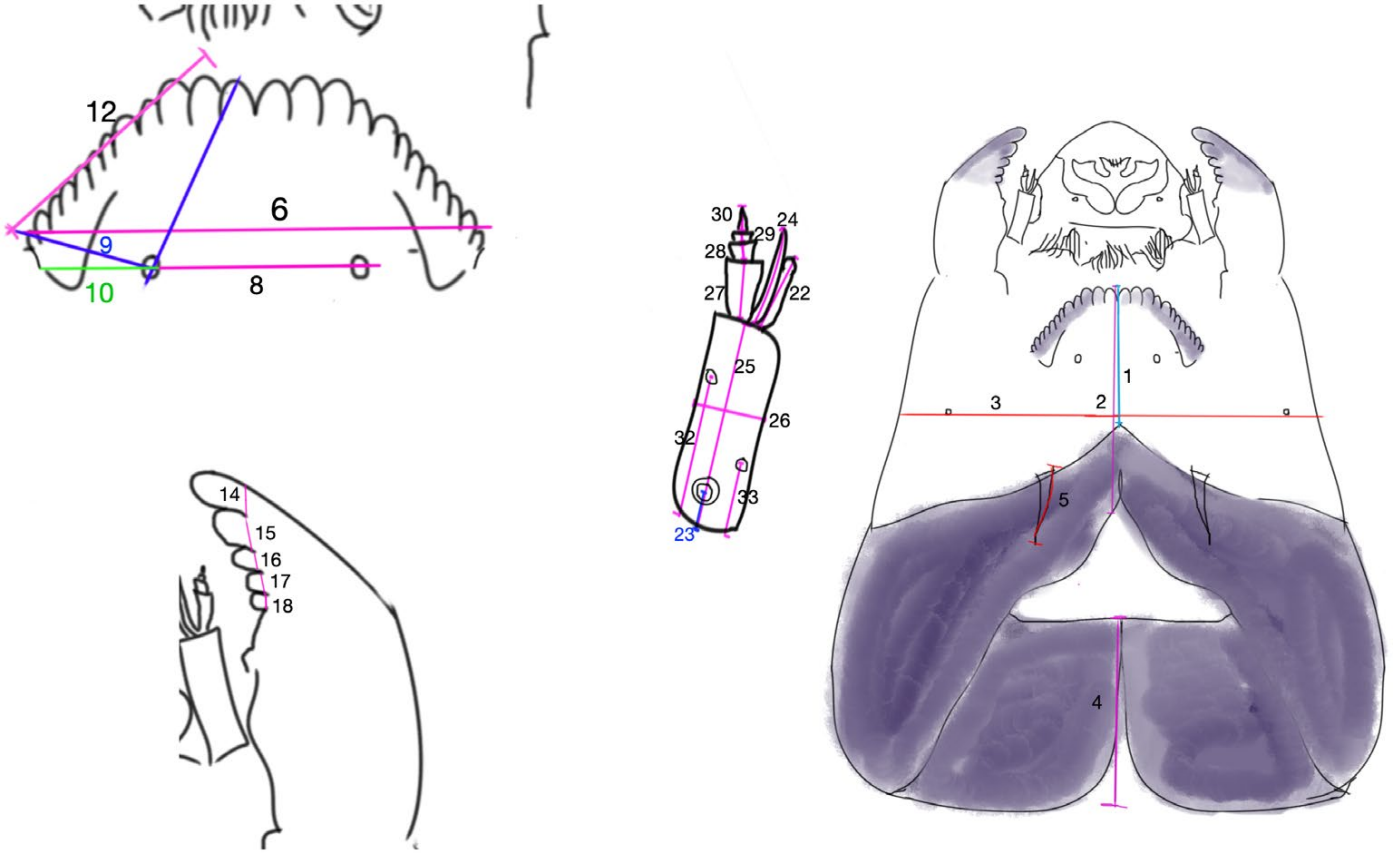
Morphological measurements evaluated for the *Paraheptagyia* larvae. Head capsule: 1 Length 1: From mentum to the front post‐occipital margin. 2 Length 2: From mentum to the back post‐occipital margin. 3 Width: Measured below the seta. Post‐occipital margin: 4 Measured on the back of the post‐occipital margin. 5 Length of the fissure. Mentum: 6 Length between the tips of the last lateral tooth. 7 Length between seta submenti. 8 Distance between the last lateral tooth and seta submenti. 9 Distance between the central tooth and seta submenti. Mandible: 10 Width of the apical tooth. 11 Width of the first inner tooth. 12 Width of the second inner tooth. 13 Width of the third inner tooth. 14 Width of the fourth inner tooth. Antenna: 15 AR (antennal ratio): Length of the first segment divided by the sum of the lengths of the second to fifth segments. 16 Length of the blade. 17 Distance between the base of the antenna and the OR. 18 Length of the seta. 19 First segment length. 20 First segment width. 21 Total length. 22 Second segment length. 23 Third segment length. 24 Fourth segment length. 25 Fifth segment length.

We determined larval instars through a comprehensive morphometric analysis of all collected specimens. For this, we measured 26 characters in Limaya and 25 in *Paraheptagyia* (Figures 3 and 4). These genera were treated under different terminological frameworks, following Rossaro et al. (2022): *Limaya* groups were referred to as morphotypes, reflecting uncertain species boundaries but clear morphological differences. In contrast, the *Paraheptagyia* group was considered a morphotaxon, as it includes genetically distinct lineages that are not morphologically distinguishable.

All measurements—recorded in micrometers (μm) using a Zeiss Axioscope A1 microscope with phase contrast—included the length and width of the head capsule, mentum, mandibles, and antennae for each larva. Notably, the frequency distribution of head capsule widths (Figures 3 and 4) revealed distinct, non‐overlapping size classes, clearly indicating different developmental instars. This approach for instar determination in chironomid midges is well‐established and has been applied in previous studies (Prat et al. 2024, and references therein). This allowed for unambiguous differentiation between the third and fourth larval instars. For the purpose of this study, we selected only larvae unequivocally identified as fourth‐instar based on these established morphometric criteria for all downstream analyses.

To construct the morphological matrices, we used the morphological characters of larvae used by Brundin (1966) for the original descriptions. Additionally, we searched for new characters useful for *Diamesa* larval differentiation (Schmid 1993; Rossaro and Lencioni 2015). Appendices S3 and S4 present the measurements (in micrometers) for fourth‐instar larvae (15 individuals for *Limaya* and 90 for *Paraheptagyia*, respectively). It is important to note that the groups presented in these measurements and morphological analyses (both tables and appendices) corresponded to the OTUs obtained in the molecular analysis. We calculated the mean, standard deviation, maximum, and minimum values for each measurement (Tables 2 and 3).

**TABLE 2:** Statistical analysis of the morphological measurements of the cephalic capsule evaluated for *Limaya*.

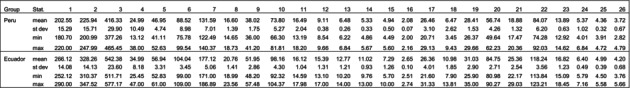

*Note:* The numbers in the heading correspond to measures shown on Figure 3.

**TABLE 3:** Statistical analysis of the morphological measurements of the cephalic capsule evaluated for *Paraheptagyia*.

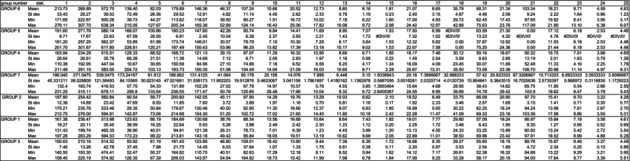

*Note:* The numbers in the heading correspond to measures shown on Figure 4. The group number refers to the classification made by bPTP.

### 2.3 Species Delimitation

We sequenced a 474 bp fragment of the cytochrome oxidase I (*COI‐5P*mtDNA) for 168 individuals (156 larvae and 12 pupae, including exuviae). To strengthen the phylogenetic framework, we incorporated *Boreoheptagyia* (Diamesinae) from GENBANK (OM302508) and *Parametriocnemus* (Orthocladiinae) from our database (OR359493) as outgroups. We used Geneious Prime 2023.0.4 (Drummond et al. 2009) to edit and assemble Diamesinae and *Parametriocnemus* raw sequences and checked for the presence of stop codons in Mesquite V. 3.70 (Maddison and Maddison 2021). We aligned the sequences with the online version of MAFFT (Katoh et al. 2019) and deposited them in GenBank under accession numbers OR359493–OR359606 and OR552540–OR552556 (Appendix S2).

We used two different methods for the delimitation of Diamesinae species to have a consensus and avoid bias related to the taxonomic rank used, the difficulty of identifying individuals by morphological characterization, the geographic distance between samples, even of the same species, and the number of haplotypes found (Zhang et al. 2013; Kapli et al. 2017; Magoga et al. 2021; Silva et al. 2023). The first method was a distance‐based approach performed using the alignment obtained with MAFFT. We used the automatic barcode gap discovery—ABGD and the assemble species by automatic partitioning—ASAP, both on the web interface, with the Kimura 2‐parameter model and the remaining parameters with default settings (Puillandre et al. 2012, 2021). The second method used was bPTP, which is a tree‐based approach (Zhang et al. 2013) implemented on the web interface, with 500,000 MCMC generations leaving the other default parameters. For this, we used the best tree obtained on Raxml v.8 (Stamatakis 2014) after a maximum likelihood (ML) analysis (1000 bootstrap and GTRCAT model) on the CIPRES Science Gateway High Performance Computing platform (http://www.phylo.org; Miller et al. 2011).

We also created a matrix of distances and a haplotype network for both genera to check for overlap in intraspecific ranges and interspecific distances. This also allowed us to visualize whether haplotypes were unique or shared between OTUs. We calculated the distance matrices in MEGA11: Molecular Evolutionary Genetics Analysis version 11 (Tamura et al. 2021), using K2P as the substitution model standard for *COI‐5P*mtDNA gen. For haplotype networks, we used the median‐joining (MJ) method (Bandelt et al. 1999) as implemented in PopART v1.7 (Leigh and Bryant 2015) and DnaSP6 (Rozas et al. 2017). We chose this method for its suitability in visualizing intraspecific and shallow interspecific genetic relationships, offering several advantages pertinent to our species delimitation study.

## 3 Results

We found Diamesinae at 20 sampling sites and collected 190 individuals (177 larvae, 3 pupae, and 10 pupal exuviae). Of these, 28 larvae and 1 pupal exuviae belonged to the genus *Limaya*, while 149 larvae, 2 pupae, and 9 pupal exuviae were from *Paraheptagyia*. While *Limaya* was present only at some sampling sites in Peru and Ecuador, *Paraheptagyia* showed a larger distribution across all the mountain ranges studied (Table 1). The study found that both genera exhibited no preference for either the Andean forest or the páramo ecosystem. The physicochemical variables exhibited considerable variation between all sampling sites, with ranges spanning 3.9–8.06 for pH, 13.7–241.9 for conductivity, and 16.18–1184.22 for flow rate. In addition, the resulting values of the IHF and QBR‐And indices, which ranged from 51 to 87 for the IHF (≥ 60 adequate, 40–60 limited) and 70 to 100 for the QBR‐And (excellent ≥ 96, good 76–95, or medium 51–75), indicated that both genera were associated with heterogeneous habitat and a riparian forest classified mostly as good according to Acosta et al. (2009) (see Appendix S1).

*Paraheptagyia* larvae inhabited rivers flowing into both the Atlantic and Pacific Oceans, at altitudes ranging from 2457 m a.s.l. in Ecuador to 4486 m a.s.l. in Peru. In contrast, we found *Limaya* exclusively in rivers draining into the Pacific Ocean, where it occupied a more restricted elevation range (3278–3987 m a.s.l.) than *Paraheptagyia*. *Paraheptagyia* individuals exhibited a broader temperature range (4.6°C–13.6°C), while *Limaya* individuals showed a narrower range (6.6°C–11.8°C). This pattern held true for pH, with *Paraheptagyia* tolerating a broader range (3.9–8.06) than *Limaya* (7.34–8.06).

### 3.1 Morphological Analysis

Our morphological analysis of 15 fourth‐instar *Limaya* larvae revealed consistent size trends that suggest the presence of two morphotypes, one from Ecuador and another from Peru. Ecuadorian specimens showed higher mean values across all measured characters compared to Peruvian specimens. For example, the antennal ratio (AR) averaged 2.67 ± 0.10 in Ecuadorian larvae versus 2.08 ± 0.07 in Peruvian larvae (Table 2). We could not recognize any morphotaxon within the *Paraheptagyia* larvae (90 individuals), despite our efforts to identify new morphological characters (Table 3, Appendix S4). The pupae belonged to the *Paraheptagyia cinerascens* group, which includes *P. cinerascens* , *P. nitescens*, and *P. andina* . However, it was not consistent with *P. andina* , as our pupae exhibited an inner spur of the hind tibia that was nearly twice as long as the tibial diameter. In contrast, *P. andina* has a length that is only slightly longer than the apical diameter of the tibia (Brundin 1966; Table 3).

Not all larvae were in the fourth‐instar stage, and the mounting quality varied. Consequently, we could not take all measurements for every individual, as Appendices S3 and S4 show. Some individuals were too damaged for measurement. The most common issue across both genera was a bent or broken mentum. Additionally, *Paraheptagyia* lacked some antennal segments, and *Limaya* missed its post‐occipital margin. This limitation posed problems in using several characters to distinguish morphotypes.

### 3.2 Molecular Taxonomy

Out of 190 specimens analyzed, we successfully amplified 168, yielding 130 high‐quality sequences (Appendix S2). We obtained 129 sequences from larvae (17 *Limaya* and 112 *Paraheptagyia*) and one from a *Paraheptagyia* pupa. To facilitate comparison across analyses, all OTUs were consistently color‐coded across the ML trees, haplotype networks, and distance matrices.

**FIGURE 5:**
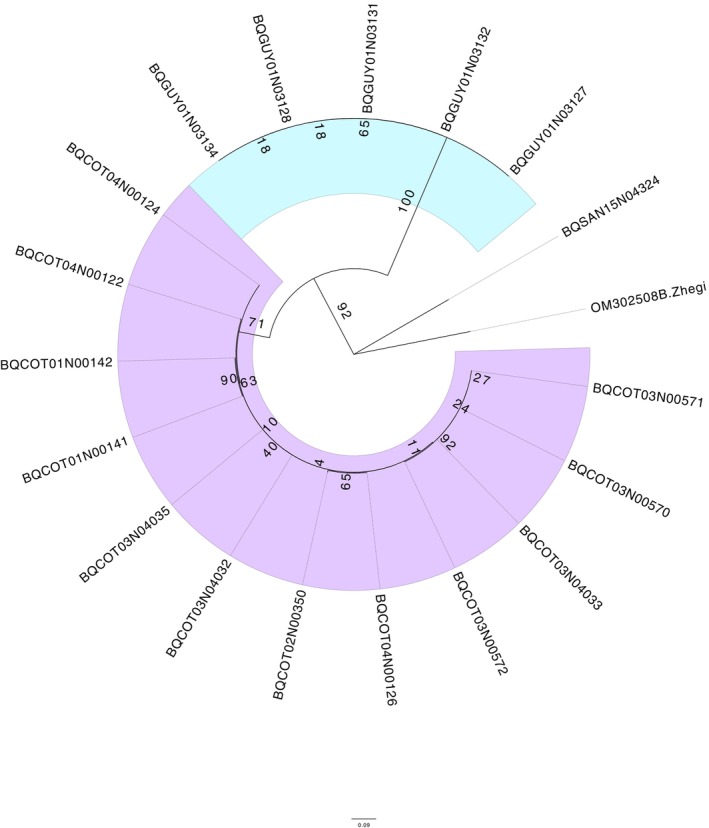
Maximum likelihood (ML) tree for the genus *Limaya* (RA × ML). Bootstrap support values are displayed at each node. Branch coloring highlights the two molecular operational taxonomic units (OTUs), which are congruent with the two distinct morphotypes identified for *Limaya*. Colors also correspond to their geographic origin: The Peruvian lineage is colored purple, and the Ecuadorian lineage is colored blue.

**FIGURE 6:**
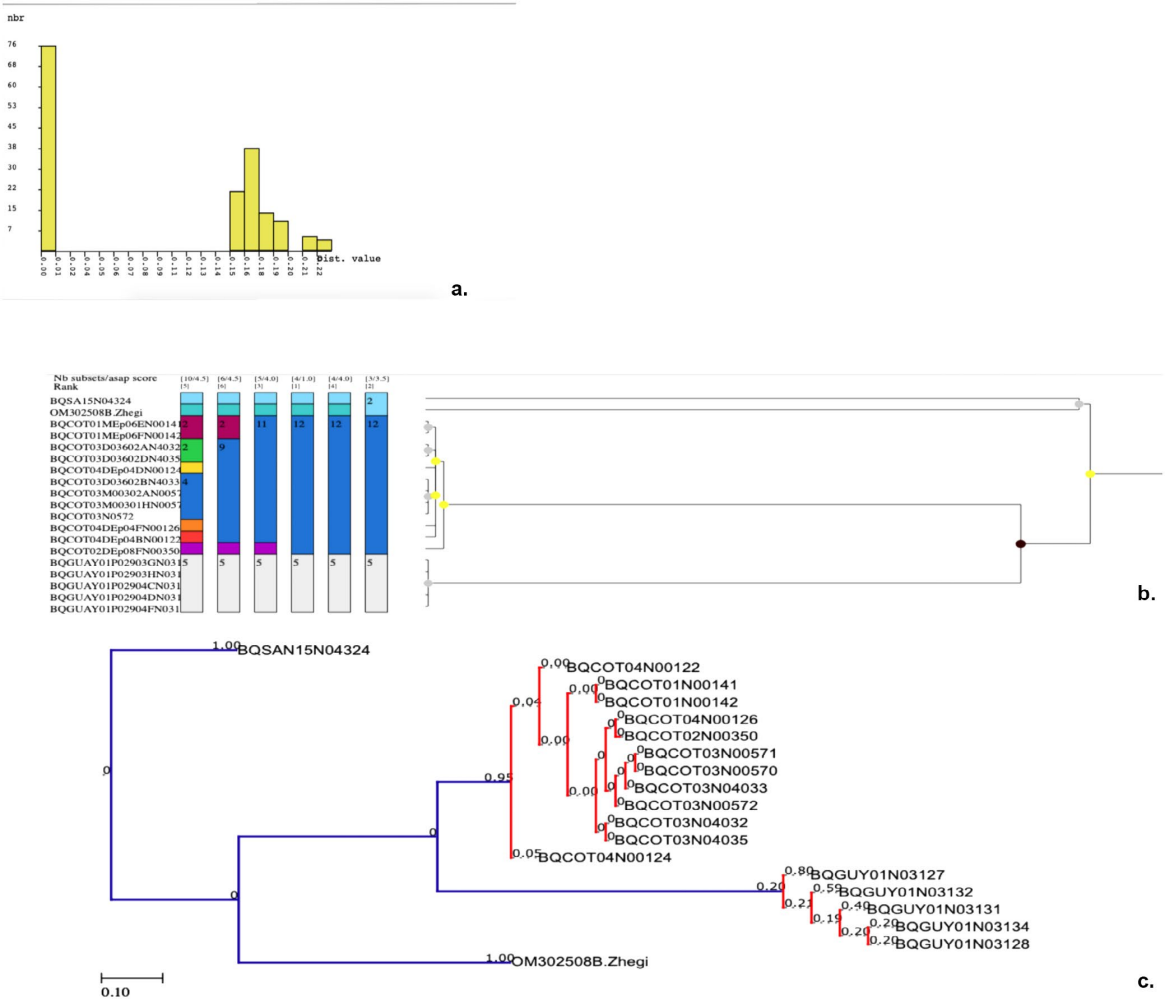
Species delimitation results for *Limaya*. (a) Histogram showing the barcode gap analysis. (b) Box subset graph from ASAP analysis. (c) Maximum Likelihood tree with bPTP species delimitation results overlaid.

The species delimitation revealed two different OTUs in the genus *Limaya* using the ML tree (Figure 5) and the three different methods, the ABGD, the ASAP, and the bPTP (Figure 6). These results aligned consistently with the morphological characterization, which also indicated two distinct morphotaxa. The ABGD frequency histogram showed the barcode gap between 0.01 and 0.15, identifying two OTUs, one from Ecuador and another from Peru. The ASAP method yielded equivalent results with a *p*‐value of 0.001, an ASAP score of 1.00, and the same barcode gap (0.01 to 0.15). Similarly, in bPTP, *Limaya* was also split into an Ecuadorian and a Peruvian group.

**FIGURE 7:**
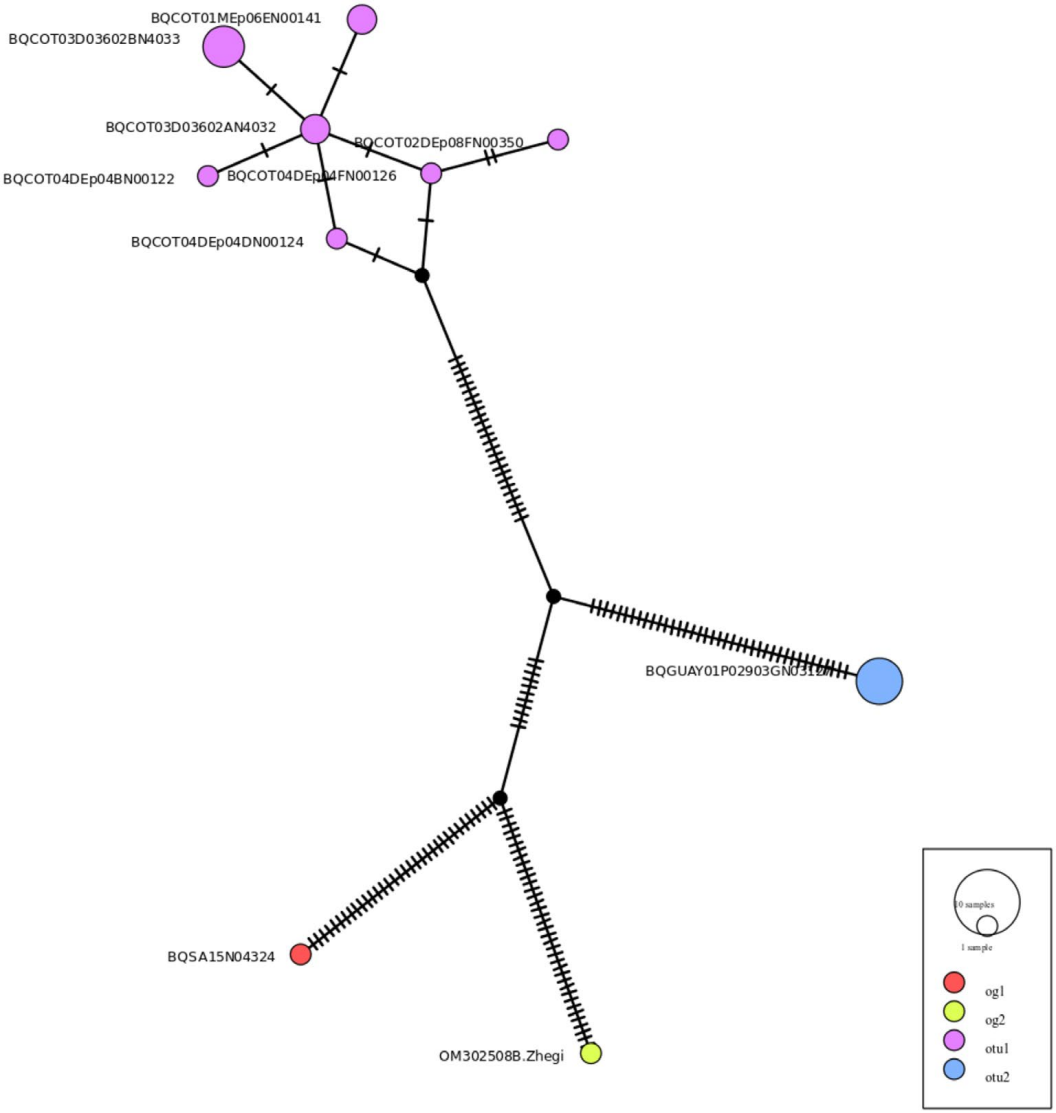
Haplotype median‐joining network for the genus *Limaya*. Each circle represents a unique haplotype, with its diameter proportional to haplotype frequency. The Peruvian group is colored purple, and the Ecuadorian haplotype is colored blue. Dashes on the branches represent single mutational steps between haplotypes. Small uncolored circles represent hypothetical or unsampled intermediate haplotypes.

The distance matrix analysis for *Limaya* (Table 4) revealed distinct patterns consistent with the delimitation of two separate OTUs from Ecuador and Peru. Intraspecific distances were notably low (0% for Ecuador; 0.9% for Peru in terms of nucleotide divergence), exhibiting no overlap with the interspecific divergence of 15.9% between these two lineages. Complementing this, the haplotype network (Figure 7) clearly displayed four distinct groups: the two designated outgroups, a single dominant haplotype representing the Ecuadorian OTU, and a clustered group of haplotypes for the Peruvian OTU. These two ingroup OTUs were separated by numerous mutational steps, aligning with the high interspecific divergence observed in the distance matrix.

**TABLE 4:** Summary of COI‐5P genetic distance statistics for *Limaya* operational taxonomic units (OTUs).

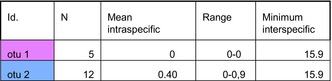

*Note:* This section provides for each delimited OTU its identification (id), the number of organisms (*N*), the mean intraspecific genetic distance, the range of intraspecific distances, and the minimum interspecific genetic distance.

**FIGURE 8:**
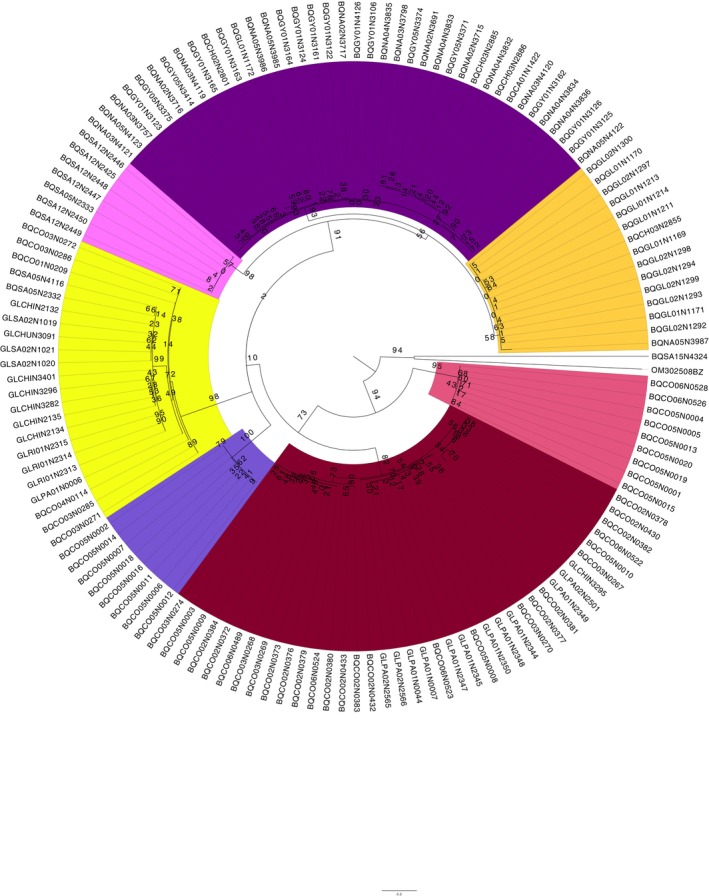
Maximum likelihood (ML) tree for the genus *Paraheptagyia* (RA × ML). Bootstrap support values are displayed at each node. Branch coloring indicates distinct molecular clades within the genus, which align with the operational taxonomic units (OTUs) delimited by the bPTP method. Each color represents a putative species or lineage as identified by this delimitation approach. The individual highlighted in blue represents the single pupa identified based on morphological characters.

**FIGURE 9:**
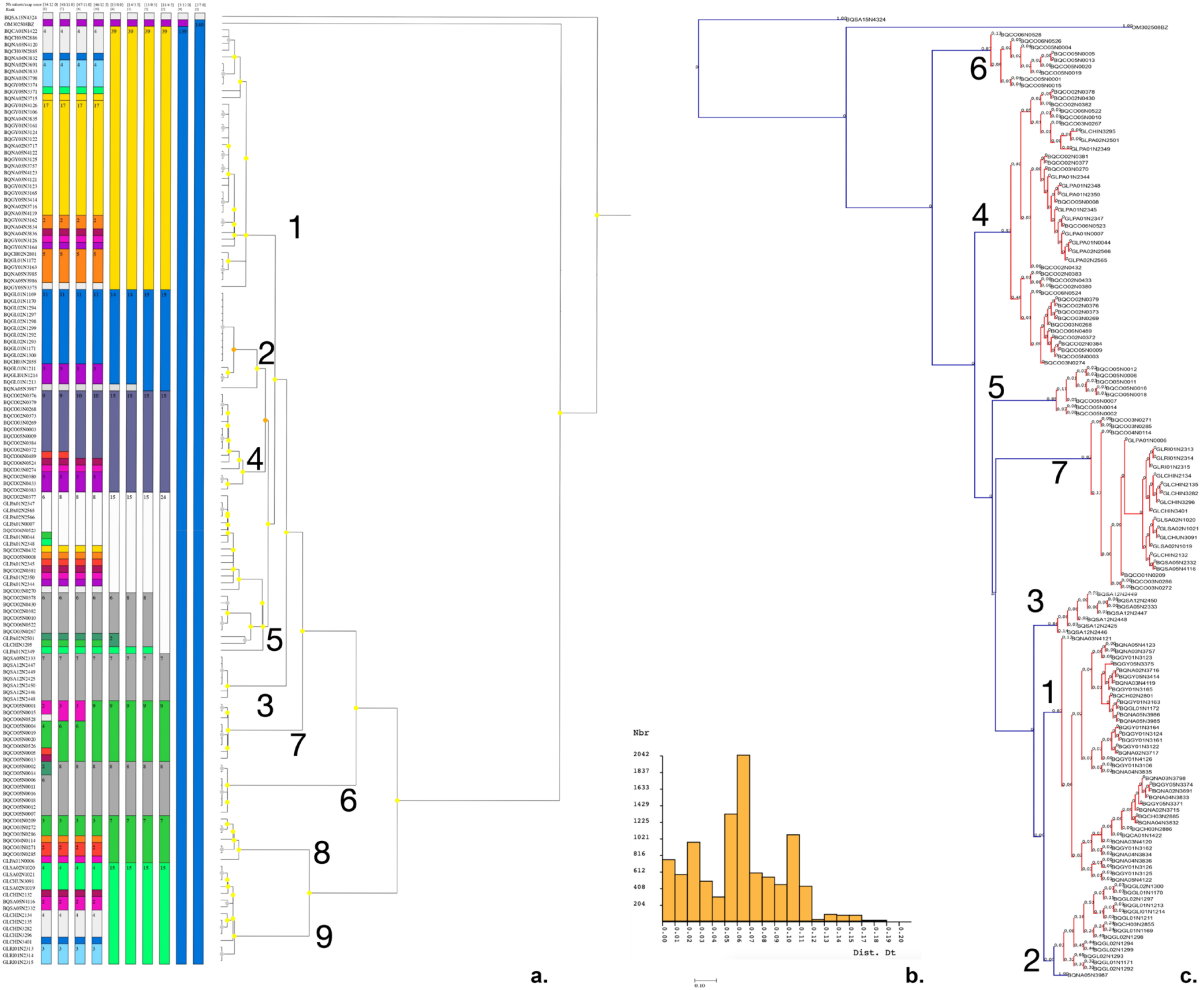
Species delimitation results for *Paraheptagyia*. (a) Histogram showing no barcode gap. (b) Box subset graph from ASAP analysis. (c) Maximum Likelihood tree with bPTP species delimitation results overlaid.

For *Paraheptagyia*, the ML tree (Figure 8) and both methods yielded comparable results. However, while ML and bPTP identified seven distinct OTUs, ABGD and ASAP identified nine (Figure 9). Both methods largely agreed on the delimitation of certain lineages. The distinction lay in the fact that ASAP and ABGD divided the two large groups from Peru into four distinct OTUs (group 4–5 and group 8–9 in Figure 9). However, the ML tree showed relatively short branches, the distance methods did not demonstrate a clear barcode gap, and the ASAP score was 4.50, which was not low enough. Analysis of the *Paraheptagyia* distance matrices (Table 5a,b) revealed patterns that corresponded with the subsequent species delimitations from ASAP and bPTP. Specifically, some OTUs exhibited overlapping maximum intraspecific distances with minimum interspecific distances, indicating an absence of a clear barcode gap. Conversely, other OTUs showed clear separation based on distance, although the minimum interspecific distances between them remained very low, ranging from 1.6% to 4.9% nucleotide divergence.

**TABLE 5:** Summary of COI‐5P genetic distance statistics for *Paraheptagyia* operational taxonomic units (OTUs). a. Following bPTP delimitation. b. Following ASAP delimitation.

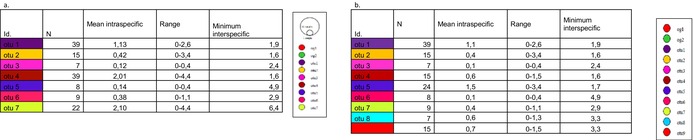

*Note:* This section provides for each delimited OTU its identification (id), the number of organisms (*N*), the mean intraspecific genetic distance, the range of intraspecific distances, and the minimum interspecific genetic distance.

**FIGURE 10:**
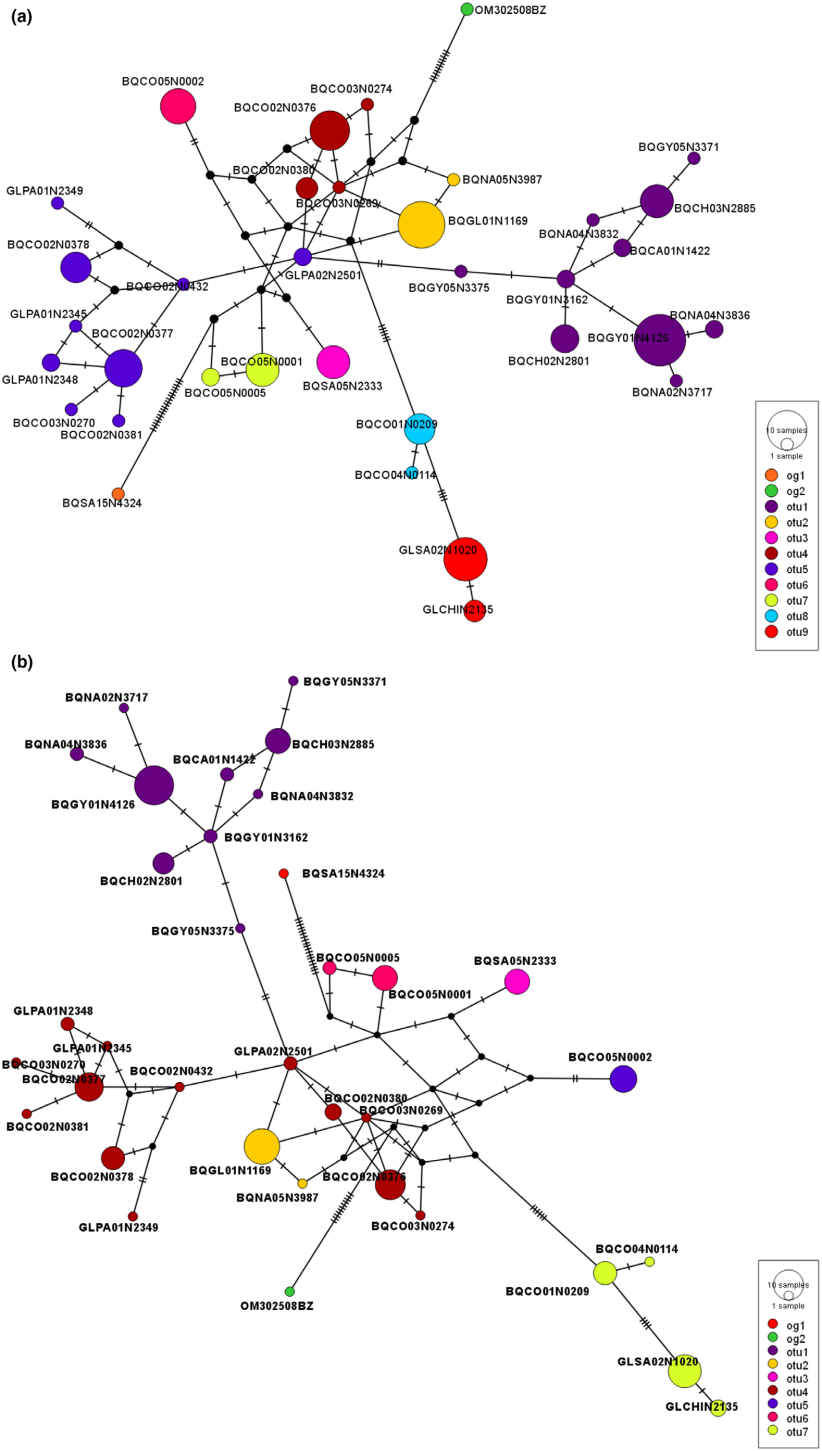
Haplotype median‐joining networks for the genus *Paraheptagyia*. (a) Network based on ASAP species delimitations. (b) Network based on bPTP species delimitations. In both networks, each circle represents a unique haplotype, with its diameter proportional to haplotype frequency (number of individuals). Colors indicate the operational taxonomic units (OTUs) as delimited by the respective method. Dashes on the branches represent single mutational steps between haplotypes. Small uncolored circles represent hypothetical or unsampled intermediate haplotypes.

We constructed and colored the haplotype networks for *Paraheptagyia* based on ASAP and bPTP delimitations (Figure 10a,b) to provide a detailed visual representation of genetic clustering. Both ASAP and bPTP delimited several clear genetic clusters (e.g., OTUs 1–9 in ASAP and OTUs 1–7 in bPTP), with each OTU assigned a unique color and no haplotype shared between different clusters.

Some differences emerged between the two methods. For example, bPTP's OTU 4 included both OTUs 4 and 5 from the ASAP analysis, while bPTP's OTU 7 corresponded to ASAP's OTUs 8 and 9. In the bPTP‐colored network, OTU 4 formed a broad cluster but displays internal structure: it included a central core corresponding to ASAP's OTU 4 and a more peripheral portion that matched ASAP's OTU 5.

Notably, the haplotype networks revealed a set of central and highly connected haplotypes that acted as hubs in the structure. In both the ASAP and bPTP networks, the central connectivity was largely driven by Peruvian lineages. In the bPTP network, OTU 4 (a southern Peruvian group) occupied the central position and connected, directly or through short mutational steps, all other OTUs. In the ASAP network, this same lineage split into OTUs 4 and 5, but the central structure was preserved: a single haplotype from OTU 5—corresponding to the core of bPTP's OTU 4—directly linked to OTUs 1, 2, and 4, while OTU 4 connected OTUs 6, 8, and 9. Additional inferred haplotypes connected this central region to OTUs 3 and 7. This pattern suggests a consistent signal of a Peruvian origin for the radiation, regardless of the delimitation method used. Whether represented as a single OTU (in bPTP) or split into two clusters (in ASAP), the central Peruvian haplotypes served as the main hub from which the remaining OTUs radiate outward.

All the molecular data indicated that *Paraheptagyia* individuals can be distinguished by their place of origin. Individuals from Peru emerged in different branches and were distinct from individuals from Colombia and Ecuador. This geographic separation was consistent across most delimitation methods, with the only exception being the genetic distance matrix, where some levels of intra‐ and inter‐group distances partially overlapped. The successful amplification of the pupa classified as part of the de *P. cinerascens* group (except for *P. andina* ) substantiated the inclusion of individuals closely related to it within this group, as evidenced by the association of stages (Figure 8).

## 4 Discussion

### 4.1 Taxonomic Challenges

There is an obvious lack of studies and researchers focusing on groups with challenging taxonomy, such as Chironomidae, especially when these organisms inhabit high‐altitude regions in countries where previous research is scarce (Brundin 1966; Roback and Coffman 1983; Spies and Reiss 1996). While ecological studies in these areas often include larvae and pupae, adults are typically underrepresented because they are less frequently captured due to the prevailing low temperatures and their reduced flight activity (Pinilla‐Agudelo 2000; del Carmen Zúñiga and Cardona 2009). In fact, adult captures tend to be abundant only when using artificial light or sticky traps (Ríos‐Touma et al. 2012). Larvae, in contrast, represent the life stage most frequently encountered and collected during aquatic surveys, making them indispensable for comprehensive studies on the distribution, biodiversity, and ecology of these groups. They provide the most accessible data for taxonomic and biogeographical analyses. Moreover, since it is sometimes impossible to delimit species based solely on morphology, larval barcoding has become a valuable tool for investigating biodiversity, speciation, and the biogeographic distribution of diverse midge species or OTUs (Ekrem et al. 2007, 2010; Silva et al. 2023).

### 4.2 Integrating Morphological and Molecular Data

In this study, we applied both morphological and molecular tools to identify and delimit species within *Limaya* and *Paraheptagyia*. Our morphological analysis of *Limaya* fourth‐instar larvae revealed consistent overall size differences between the individuals from Ecuador and Peru. Specifically, Ecuadorian specimens showed larger mean values across all measured morphometric characters compared to Peruvian specimens. While some minimum and maximum values for certain characters occasionally overlapped, the robust differences in their mean measurements, combined with the established importance of characters such as AR as a widely used diagnostic character in Diamesinae taxonomy (Rossaro and Lencioni 2015; Montagna et al. 2016), enabled us to confidently differentiate these Ecuadorian and Peruvian larvae. These two morphologically delimited morphotypes for *Limaya* were perfectly congruent with our molecular findings, as our molecular results analyses consistently recovered two distinct molecular OTUs across the ML tree, the distance matrix, the haplotype network, and all three species delimitation methods (ABGD, ASAP, and bPTP). This congruence highlights the effectiveness of combining both approaches for species delimitation in this genus (Montagna et al. 2016; Stur et al. 2019; Lencioni et al. 2021; Makarchenko et al. 2023; Mrozińska and Obolewski 2024). Unfortunately, despite the larvae of these two morphotypes fitting the original description, it was not possible to name them or associate them with the species described by Brundin (1966), as there is no mention of size or measurements. However, the molecular data of our work will be useful in future barcoding studies of adults.

In contrast to *Limaya*, our analysis did not successfully differentiate morphotaxa in *Paraheptagyia* larvae. Despite thorough examination, the 26 morphological character measurements among the molecularly defined OTUs for *Paraheptagyia* showed no differences, preventing the establishment of reliable morphological distinctions. This challenge in differentiating *Paraheptagyia* larvae is a common issue within Chironomidae. In many cases, genetically distinct species lack clear morphological differences (cryptic diversity) or exhibit minimal morphological change over evolutionary time (morphological stasis), making identification difficult even after exhaustive searches for new characters (Cranston and Krosch 2011; Prat et al. 2013; Lin et al. 2018). For this reason, many ecological and biomonitoring studies rely on larval morphotaxa to understand the relationships between environmental parameters and coexisting taxa (Rossaro et al. 2022). Moreover, recent work emphasizes the importance of integrating morphotype‐based approaches with molecular data to improve ecological interpretations and support more robust biodiversity assessments (Lencioni et al. 2021; Mrozińska and Obolewski 2024; Silva et al. 2023).

Despite the morphological challenges, our molecular data for *Paraheptagyia* presented a complex picture, with the ML tree and bPTP identifying seven OTUs, whereas ABGD and ASAP detected nine. Species delimitation for *Paraheptagyia* proved particularly intricate due to the absence of a clear ‘barcode gap’ in the genetic distance matrices and the ASAP method. This has been reported for several groups in Chironomidae; usually, different methods yield different numbers of OTUs (Montagna et al. 2016; Silva et al. 2023; Yao et al. 2025). Specifically, the maximum intraspecific genetic distance for some putative OTUs was observed to exceed the minimum interspecific distance for other OTUs. This overlap makes it extremely challenging to use simple distance thresholds reliably for species identification. However, for the OTUs where distance matrices showed an overlap between intra‐ and interspecific divergence (i.e., those with an absent barcode gap), the haplotype networks clearly revealed them as distinct and well‐separated genetic clusters. This visual evidence from the networks, coupled with the delimitations from the model‐based methods, supports their interpretation as evolutionarily independent lineages, despite the lack of a discrete distance gap (Song et al. 2016; Yao et al. 2025). This suggests that the absent barcode gap (Figure 9), along with the relatively short branches in the ML tree (Figure 8) and the presence of multiple molecular OTUs, might be attributed to factors such as incomplete lineage sorting and recent divergence, rather than a lack of true species‐level differentiation. While the ML tree (Figure 8) suggested certain relationships (e.g., OTUs 1 and 2 closer to OTU 3), the haplotype network (Figure 10) provided a more detailed population‐level perspective, highlighting direct connections between southern Peruvian lineages (OTU 4 and haplotype of OTU 5) and northern ones (OTUs 1 and 2), consistent with recent dispersal or ongoing gene flow. The combination of these analytical approaches suggests that the speciation process for these lineages might still be ongoing (Wiemers and Fiedler 2007; Silva et al. 2023; Yao et al. 2025).

By applying molecular tools to *Paraheptagyia*, we were able to confidently confirm that the pupa (highlighted in blue in Figure 8) and the larvae close to it belong to the same group, *P. cinerascens* , excluding *P. andina*. This successful association of life stages, as demonstrated by previous studies (Brundin 1966; Ekrem et al. 2007, 2010; Lin et al. 2018), proved invaluable in clarifying taxonomic relationships and confirming species identity, particularly in groups where larval morphology alone is insufficient. Indeed, several authors reported success using *COI‐5P*mtDna to identify and delimit cryptic species with interesting results (Montagna et al. 2016; Lin et al. 2018; Stur et al. 2019; Gadawski et al. 2022; Mrozińska and Obolewski 2024).

### 4.3 Distribution and Biogeography

Despite the challenges in morphological differentiation for *Paraheptagyia*, molecular analyses for both *Paraheptagyia* and *Limaya* consistently showed strong genetic differentiation by place of origin, with Peruvian OTUs clearly distinct from those in Ecuador and Colombia. For *Paraheptagyia*, this geographical differentiation was exclusively evident through molecular data. In contrast, for *Limaya*, this molecular geographic distinction was further corroborated by the consistent morphological differences observed between specimens from different localities.

We hypothesize this pronounced genetic partitioning, particularly between southern and northern lineages, is strongly influenced by the Huancabamba Depression, a significant geological downwarp in the Andes situated between northern Peru and southern Ecuador. This depression acts as an important natural barrier to gene flow for various species, mainly due to its unique geographic and ecological characteristics. Its characteristics prevent the free movement of many high‐altitude, cold‐adapted organisms that live in fast‐flowing rivers (Weigend 2002; Weigend et al. 2010; Acosta and Prat 2018; Hazzi et al. 2018). As a consequence, populations of these species are isolated on one or both sides of the depression, limiting their ability to interbreed and exchange genetic material, leading to distinct evolutionary trajectories and possible speciation events over time. This isolation effect is well documented not only for the target genera, but also for other midges, such as *Barbadocladius* (Orthocladiinae; Prat et al. 2013), *Polypedilum* (Chironominae; Ballesteros et al. 2022), and Podonominae (Acosta and Prat 2018), and other invertebrates, such as the mantis genus *Pseudopogonogaster* (Rivera et al. 2011), which exhibit similar distribution patterns restricted to specific habitats. These distribution limitations underscore the role of the depression as a selective force shaping biodiversity by restricting both species range and gene flow.

Our findings for *Limaya* corroborate its previously reported distribution range, extending as far north as Ecuador (Prat et al. 2018), beyond earlier reports of its presence only in Peru (Brundin 1966; Roback and Coffman 1983; Ruiz‐Moreno et al. 2000). Interestingly, while the holotype (male) and additional material for *Limaya* were obtained from rivers flowing into the Atlantic Ocean in Argentina and Chile (Pacific Ocean), our study exclusively found *Limaya* species in rivers flowing into the Pacific Ocean. This suggests that the absence of *Limaya* in samples from the high Andean basins of the Atlantic slope warrants further research and dedicated sampling efforts.

For *Paraheptagyia*, its broader reported distribution (Brundin 1966; Roback and Coffman 1983; Ruiz‐Moreno et al. 2000; Prat et al. 2018) suggests a wider range of tolerance to altitude, pH, and temperature compared to *Limaya*. This broader tolerance might explain why *Paraheptagyia* has been found as far north as Colombia despite the presence of barriers like the Huancabamba Depression, whereas *Limaya* has not extended as far north in our samples. Given the abundance of *Paraheptagyia* individuals we found, its absence further north beyond reported ranges raises questions for future investigations.

**FIGURE 11:**
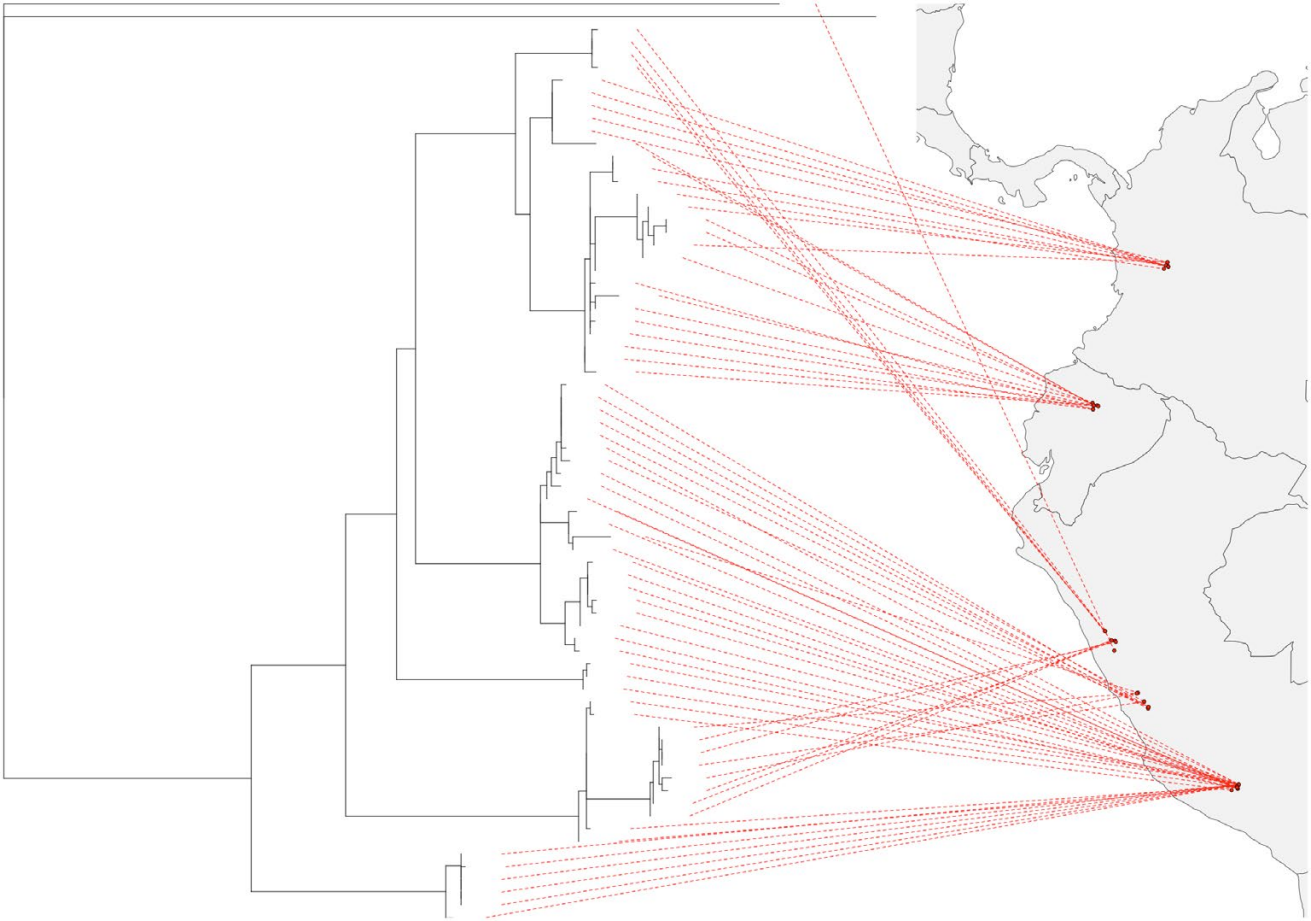
Geographic projection of the maximum likelihood tree for *Paraheptagyia* on the map of South America. This projection visually represents the inferred dispersal patterns, with longer branches (indicating older or more diversified lineages) predominantly observed in southern regions, and shorter branches (suggesting more recent divergences or northward dispersal) found toward the north.

By examining the ML tree of *Paraheptagyia* and its geographical overlap with the South American map (Figure 11), we can propose some tentative hypotheses. Based solely on the observation that the branches containing the Peruvian species are notably longer than those containing the species from Ecuador and Colombia, it is plausible to support Brundin's austral pattern hypothesis (Brundin 1966). This hypothesis suggests that several species of Chironomidae entered South America from the southern regions and subsequently dispersed northwards. The longer branch lengths associated with Peruvian species could imply older divergence events or more significant evolutionary changes in comparison to their northern counterparts, possibly aligning with this south‐to‐north dispersal route. However, further analyses, such as molecular dating and biogeographical reconstructions, are required to substantiate this hypothesis and clarify the evolutionary history of *Paraheptagyia* in the context of South American biogeography (Cranston et al. 2010; Krosch et al. 2011; Semenchenko et al. 2024).

Our study focused on the biodiversity and distribution of these two genera of Diamesinae in high‐altitude streams of the Andes, from southern Peru to northern Colombia, using larval barcoding revealing a clear geographic structuring of OTUs, suggesting that historical and ecological factors may have influenced their current distribution. In conclusion, our results support two hypotheses: first, that the Andes may be promoting speciation, and second, that more species of this subfamily are found in the south than in the north due to Gondwanan dispersal. Haplotype network analyses provided crucial visual insights consistent with these patterns. For *Paraheptagyia*, the networks showed southern Peruvian OTUs (e.g., OTU 4) occupying central positions and directly connecting to both other Peruvian lineages and to the more northern Colombian and Ecuadorian OTUs. This pattern, coupled with the relatively longer branches observed for Peruvian clades in the ML tree (Figures 8 and 11), lends support to the hypothesis of a south‐to‐north dispersal or radiation originating from southern Peruvian lineages. Furthermore, the networks clearly depicted distinct, geographically separated clusters of haplotypes, consistent with the isolating effect of barriers like the Huancabamba Depression.

The next step would be to determine whether the OTUs of both genera found in the northern regions of South America reached this area due to specific geographical conditions—such as their migration before the formation of the Huancabamba Depression (Cretaceous, Mourier et al. 1988; Mitouard et al. 1990)—or during a glacial process at the end of the Pleistocene (Vuilleumier 1971; Nevado et al. 2018). Another possibility is that these OTUs managed to spread northwards because they had a higher tolerance to warmer temperatures (Gavashelishvili and Tarkhnishvili 2016; Acosta and Prat 2018). Further investigation into these potential factors will be crucial to understanding the mechanisms driving both *Limaya* and *Paraheptgayia*'s current distribution.

## Author Contributions

**Diana C. Hoyos Jaramillo:** conceptualization (lead), data curation (lead), formal analysis (lead), investigation (equal), methodology (equal), visualization (lead), writing – original draft (lead), writing – review and editing (equal). **Raúl Acosta:** funding acquisition (supporting), investigation (lead), methodology (equal), validation (equal), writing – review and editing (equal). **Carles Ribera:** data curation (supporting), methodology (supporting), writing – review and editing (equal). **Núria Bonada:** conceptualization (equal), validation (equal), writing – review and editing (equal). **Narcís Prat:** conceptualization (equal), data curation (supporting), funding acquisition (lead), methodology (equal), project administration (lead), validation (equal), writing – review and editing (equal).

## Conflicts of Interest

The authors declare no conflicts of interest.

## Supporting information


**Data S1:** ece372333‐sup‐0001‐DataS1.xlsx.

## Data Availability

The sequences used in this study have been deposited in GenBank under accession numbers OR359493–OR359606 and OR552540–OR552556 (Appendix S2). The morphological data can be found in the appendixes. Should further information or data be required, it will be made available.
